# Loss of heterozygosity and *SOSTDC1 *in adult and pediatric renal tumors

**DOI:** 10.1186/1756-9966-29-147

**Published:** 2010-11-16

**Authors:** Kimberly R Blish, Kathryn A Clausen, Gregory A Hawkins, A Julian Garvin, Mark C Willingham, Julie C Turner, Frank M Torti, Suzy V Torti

**Affiliations:** 1 Department of Molecular Medicine, Wake Forest University School of Medicine, Medical Center Boulevard, Winston-Salem, NC 27157, USA; 2 Department of Cancer Biology, Wake Forest University School of Medicine, Medical Center Boulevard, Winston-Salem, NC 27157, USA; 3 Center for Genomics and Personalized Medicine Research, Wake Forest University School of Medicine, Medical Center Boulevard, Winston-Salem, NC 27157, USA; 4 Department of Pathology, Wake Forest University School of Medicine, Medical Center Boulevard, Winston-Salem, NC 27157, USA; 5 Comprehensive Cancer Center, Wake Forest University School of Medicine, Medical Center Boulevard, Winston-Salem, NC 27157, USA; 6 Department of Biochemistry, Wake Forest University School of Medicine, Medical Center Boulevard, Winston-Salem, NC 27157, USA

## Abstract

**Background:**

Deletions within the short arm of chromosome 7 are observed in approximately 25% of adult and 10% of Wilms pediatric renal tumors. Within Wilms tumors, the region of interest has been delineated to a 2-Mb minimal region that includes ten known genes. Two of these ten candidate genes, *SOSTDC1 *and *MEOX2*, are particularly relevant to tumor development and maintenance. This finding, coupled with evidence that SOSTDC1 is frequently downregulated in adult renal cancer and regulates both Wingless-Int (Wnt)- and bone morphogenetic protein (BMP)-induced signaling, points to a role for SOSTDC1 as a potential tumor suppressor.

**Methods:**

To investigate this hypothesis, we interrogated the Oncomine database to examine the SOSTDC1 levels in adult renal clear cell tumors and pediatric Wilms tumors. We then performed single nucleotide polymorphism (SNP) and sequencing analyses of *SOSTDC1 *in 25 pediatric and 36 adult renal tumors. Immunohistochemical staining of patient samples was utilized to examine the impact of *SOSTDC1 *genetic aberrations on SOSTDC1 protein levels and signaling.

**Results:**

Within the Oncomine database, we found that SOSTDC1 levels were reduced in adult renal clear cell tumors and pediatric Wilms tumors. Through SNP and sequencing analyses of 25 Wilms tumors, we identified four with loss of heterozygosity (LOH) at 7p and three that affected *SOSTDC1*. Of 36 adult renal cancers, we found five with LOH at 7p, two of which affected *SOSTDC1*. Immunohistochemical analysis of SOSTDC1 protein levels within these tumors did not reveal a relationship between these instances of *SOSTDC1 *LOH and SOSTDC1 protein levels. Moreover, we could not discern any impact of these genetic alterations on Wnt signaling as measured by altered beta-catenin levels or localization.

**Conclusions:**

This study shows that genetic aberrations near *SOSTDC1 *are not uncommon in renal cancer, and occur in adult as well as pediatric renal tumors. These observations of *SOSTDC1 *LOH, however, did not correspond with changes in SOSTDC1 protein levels or signaling regulation. Although our conclusions are limited by sample size, we suggest that an alternative mechanism such as epigenetic silencing of *SOSTDC1 *may be a key contributor to the reduced SOSTDC1 mRNA and protein levels observed in renal cancer.

## Background

Renal tumors affecting both adults and children are often idiopathic in origin. The clinical presentation, disease history, and treatments of renal tumors differ between children and adults. In children, the majority of renal masses are pediatric Wilms tumors. Wilms tumor is the sixth most common malignancy of childhood, annually affecting approximately 500 children in the United States [1]. While lesions respond quite well to treatment, with an overall survival rate of 85% [2], the challenge remains to identify disease subtypes so that high risk patients are sufficiently addressed while low risk patients are not overtreated.

Compared to pediatric Wilms tumors, adult renal cancers tend to be more difficult to detect and respond more poorly to treatment. Incidence of adult renal carcinoma has increased steadily since the 1970's [3]. The most prevalent type of adult renal tumor is renal clear cell carcinoma (RCC-clear), which accounts for 80-85% of adult renal cancer cases. Less common adult lesions include papillary (5-10% of cases), chromophobe, medullary, and oncocytic (< 5%) types.

Genes found within regions of loss of heterozygosity (LOH) associated with both pediatric and adult renal cancers represent candidate tumor suppressors whose inactivation may be critical for the initiation or progression of renal cancer. In both pediatric and adult tumors, cytogenetic changes have been noted on the short arm of chromosome 7. Within Wilms tumors, these include a 10% incidence of LOH on 7p [4]. Likewise, loss of 7p, duplication of 7q, and consistent gains of chromosome 7 have been identified in adult late stage RCC-clear and RCC-papillary subtypes [5-9].

In Wilms tumors, a consensus region of LOH has been identified within 7p21 containing ten known genes, including two candidate tumor suppressor genes, mesenchyme homeobox 2 (*MEOX2*) and sclerostin domain containing 1 (*SOSTDC1) *[10]. The mesenchyme homeobox 2 protein is a transcription factor that inhibits vascular endothelial cell proliferation and angiogenesis by upregulating p21 expression and decreasing NF-κB activity [11]. *SOSTDC1 *encodes a secreted signaling modulator that is known to affect signaling by bone morphogenic proteins (BMPs) and Wingless-Int (Wnt) ligands [12-14]. Previous findings demonstrated that SOSTDC1 is abundantly expressed in the renal epithelia of the distal tubules, collecting ducts, and urothelium [15] and that it is downregulated in adult renal carcinomas [16]; however, the association between LOH at *SOSTDC1 *and adult renal cancer has not been explored.

The capacity for SOSTDC1 to regulate two key signaling pathways, BMP and Wnt, in renal cells make it of particular interest as a potential renal tumor suppressor [16]. As changes in BMP signaling have been noted in a variety of tumors [17-19], including renal tumors [20], an extracellular modulator of BMP signaling could have potential tumor suppressor roles within normal kidney epithelia. Similarly, dysregulation of the Wnt pathway often plays a role in tumorigenesis [21]. In Wilms tumors specifically, mutations have been observed in β-catenin, the main intracellular effector of classical Wnt signaling [22]. Alterations in Wnt signaling have also been implicated in adult renal carcinoma [23]. The observations that *SOSTDC1 *is located within a chromosomal region frequently disrupted in renal tumors and that the SOSTDC1 protein modifies two cell signaling pathways that are critical to renal development and function, led us to investigate the relationship between LOH at 7p and *SOSTDC1 *in adult as well as pediatric kidney tumors.

## Methods

### Cells and culture conditions

The HEK-293 (CRL-1573; human embryonic kidney), MDA-MB-231 (HTB-26; epithelial adenocarcinoma), and MCF-10A (CRL-10317; mammary epithelial) cell lines were maintained as recommended by American Type Culture Collection (ATCC).

### Collection of tissues

Approval was obtained from the Institutional Review Board at Wake Forest University for the retrieval of matched normal and tumor tissues from the Tumor Bank of the Wake Forest University Comprehensive Cancer Center. Matched normal and tumor tissues were collected for 36 adult kidney cancer patients and seven pediatric Wilms tumor patients. Information concerning the patients' primary diagnoses was collected; however, no patient identifiers were obtained. An additional 18 matched normal and Wilms tumor tissues were obtained from the Cooperative Human Tissue Network (CHTN), which is funded by the National Cancer Institute. Other investigators may have received portions of these tissue samples. Patient diagnostic and treatment information were made available for each tissue. Tissues were collected as snap frozen specimens stored at -80°C.

### Sample preparation and genomic DNA isolation

Each snap frozen tissue was sectioned on a bed of dry ice to ensure minimal thawing during sample preparation. An approximately 30-50 mg piece of tissue was cut and an adjacent piece of tissue was removed for formalin fixation and paraffin embedding for subsequent histological processing. Genomic DNA was isolated from tissue samples via homogenization in ice cold lysis buffer [10 mM Tris pH 8.0, 0.1 M ethylenediaminetetraacetic acid (EDTA), 0.5% sodium dodecyl sulfate (SDS), 100 μg/mL Proteinase K, 25 μg/mL RNAase]. Subsequent phenol-chloroform extraction was carried out as previously described [24]. Integrity and concentration of each resulting DNA sample was assessed by agarose gel electrophoresis.

### Sequencing primer design

The known coding region of *SOSTDC1 *is contained within two exons. Other potentially transcribed areas have been identified in the University of California Santa Clara Genome database [25-27]. Two of these potential exons occur upstream of the coding region and an additional exon occurs between the known coding exons for a total of five putative exons or regulatory regions at this locus (see Additional file 1). Primers were designed for direct sequencing for a total of 13 pairs of direct sequencing primers (see Additional file 2). All primers were synthesized by Integrated DNA Technologies (IDT).

### PCR amplification and direct sequencing

Each direct sequencing primer pair was used to amplify all five putative regions of interest in each normal and tumor sample via PCR. PCR was performed in 40 μL reactions using 60 ng of genomic DNA, 15 pmol of both the forward and reverse primer, 4-5U of Taq polymerase (Life Technologies), 1.5 mM MgCl_2_, 200 μM dNTPs. Depending on prior reaction optimization, general cycling conditions were: 94°C 4 min, followed by 25-30 cycles at 94°C for 1 min, T_anneal _for 1 min, and at 72°C for 1 min; and finishing with a single extension cycle at 72°C for 5 min. PCR products were purified using the Quickstep 96-well PCR purification kit (Edge Biosystems). DNA sequencing was performed using the ABI BigDye Terminator sequencing kit (Applied Biosystems, Inc.) Each 10 μL sequencing reaction contained 10-50 ng of purified PCR product, 1.5 pmoles of sequencing primer, 1 μL of BigDye Terminator mix, 1.5 μL of 5 × sequencing dilution buffer (400 mM Tris pH 9.0, 10 mM MgCl_2_) and water to volume. Cycling conditions were 94°C for 1 min; 25 cycles at 94°C for 30 sec, 50°C for 30 sec, and 60°C for 4 min; and finishing with a single 72°C extension step for 5 min. The sequencing reactions were run on an ABI 3730XL DNA sequencer and data were analyzed using Sequencher software (GeneCodes, Version 4.7).

### Loss of heterozygosity analysis

To examine the area surrounding *SOSTDC1 *for loss of heterozygosity, single nucleotide polymorphisms (SNPs) were genotyped. Fifty-one SNPs within the 2.4 Mb region with high percentages of heterozygosity (> 0.45) were chosen for analysis (HapMap) [28]. Primers for each SNP were designed for analysis on the MassARRAY system (Sequenom; see Additional file 3). All primers were synthesized by IDT. The genotyping reactions were performed with 5 ng genomic DNA from each sample.

### Immunohistochemical analysis of patient samples

Formalin-fixed, paraffin-embedded renal tissue samples analyzed for LOH were sectioned and processed for immunohistochemistry as previously described [28]. Tissues were stained with anti-β-catenin antibody (BD Transduction Laboratories) or SOSTDC1-specific rabbit antiserum [16]. Primary antibody treatments were followed by incubation with ImmPRESS anti-mouse/rabbit or anti-rabbit IgG peroxidase-conjugated secondary antibodies (Vector Laboratories) and development with 3,3'-diaminobenzidine (DAB; Vector Laboratories). Stained sections were imaged using a Zeiss Axioplan2 confocal microscope (Carl Zeiss, Inc.).

### Antibody characterization

Antibody specificity was verified in four ways (see Additional file 4). First, we verified that immunohistochemical staining of tissues was not observed in the absence of SOSTDC1 antiserum. Second, we confirmed that the antiserum detected recombinant SOSTDC1 protein. Known quantities of glutathione S-transferase (GST)-tagged SOSTDC1 protein (Novus Biologicals) were gel-resolved, transferred to nitrocellulose, and immunoblotted with SOSTDC1-specific antiserum as described previously [16]. Third, antibody specificity was confirmed by peptide competition. Cells were lysed in Triton X-100 lysis buffer [50 mM Tris pH 7.5, 150 mM sodium chloride, 0.5% Triton X-100 (Sigma)] containing Complete protease and phosSTOP phosphatase inhibitor cocktail tablets (Roche Diagnostics). After protein electrophoresis, transfer, and blocking, duplicate membranes were immunoblotted with SOSTDC1-specific antiserum in the presence or absence of the immunizing peptide (Ac-CVQHHRERKRASKSSKHSMS-OH; Biosource) at a concentration of 1 μg/mL. Protein detection then proceeded as described previously [16]. Equal protein loading was verified by immunoblotting with anti-glyceraldehyde 3-phosphate dehydrogenase (GAPDH) antibody (Fitzgerald). Fourth, we confirmed that FLAG-tagged SOSTDC1 that had been immunoprecipitated by anti-FLAG antibody (M2; Sigma-Aldrich) was detected by our antibody.

## Results

### SOSTDC1 expression levels in renal carcinoma

**Figure 1:**
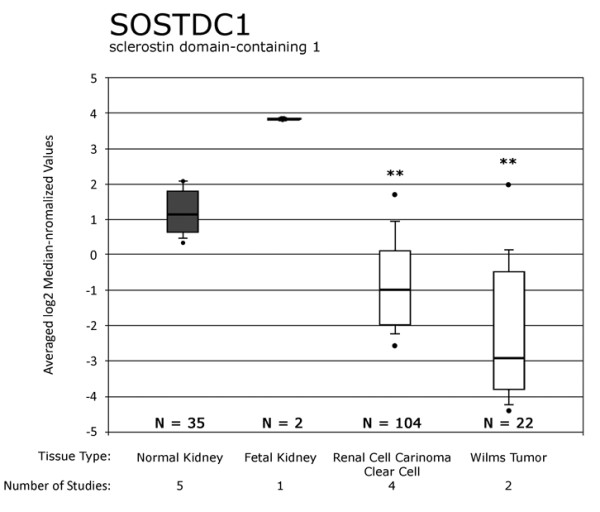
**Oncomine database shows significant SOSTDC1 downregulation in adult renal clear cell tumors and pediatric Wilms tumors**. The Oncomine database was queried for all studies involving markers in SOSTDC1 (data queried on 11/08/2010). Results of five studies were compared using the software available on the site [40-44]. Dots above and below the boxes show sample maximum and minimum values, respectively. The horizontal lines show the spread of the values from starting at the 10% value through the 90% value, with the box highlighting the range of 25% to 75%. Dark boxes show the normal or control tissues for each study and white boxes show adult clear cell renal carcinoma and Wilms tumor values. The horizontal black bar through each box shows the median value for the sample. ** p < 0.001, normal adult or fetal renal tissue compared to adult RCC or Wilms tumors.

We had previously observed that SOSTDC1 expression is decreased in adult renal carcinomas [16]. To assess whether expression levels of SOSTDC1 were similarly decreased in pediatric kidney cancer patients, we queried the Oncomine database [29]. The sample size and expression sensitivity of this method improved the likelihood of detecting a notable change in SOSTDC1 that correlated with development of renal tumors. Consistent with a potential role for SOSTDC1 as a tumor suppressor, SOSTDC1 expression was statistically significantly decreased in both adult clear cell renal carcinoma and pediatric Wilms tumors. As shown in Figure 1, there is a significant reduction in SOSTDC1 in Wilms tumors and renal clear cell carcinoma. The median value of SOSTDC1 expression in normal adult tissue was 1.13 and that in normal fetal tissue was 4.00, while the levels of SOSTDC1 expression in adult renal clear cell carcinoma and pediatric Wilms tumors were significantly lower, at -1.00 and -2.92, respectively (p < 0.001).

### Loss of heterozygosity at 7p21 within pediatric Wilms tumors

**Figure 2:**
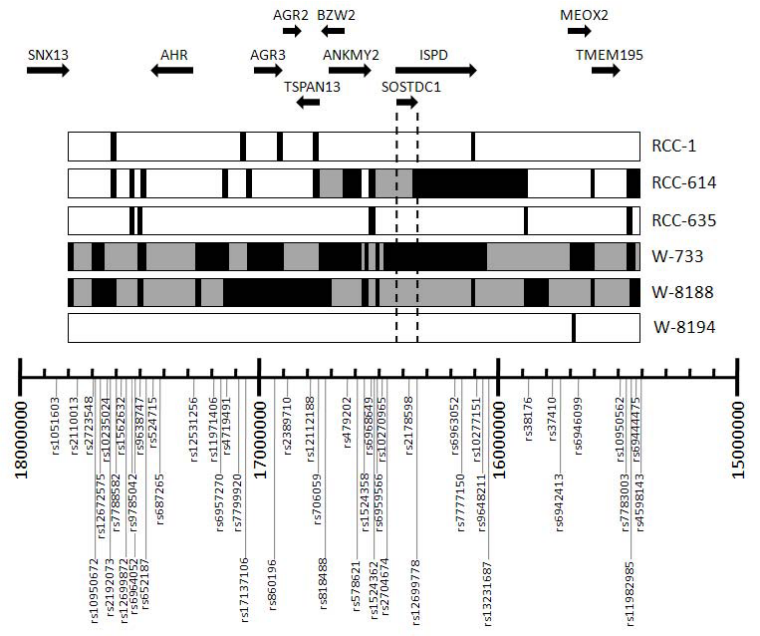
**LOH analysis in 2.4 Mb region of chromosome 7p**. Results from LOH-containing pediatric Wilms (W) and adult renal carcinoma (RCC) samples are aligned with a 7p21.1 to 7p21.2 SNP map. Patient identifiers are shown on the right; RCC denotes adult renal cell carcinoma and W denotes Wilms tumors. Only those patients exhibiting LOH are shown. The 51 SNP markers used in this study are shown along the bottom. They are mapped according to their physical location from 15400000 to 18000000 on chromosome 7p21. The terminal location is at the right; the centrosomal end is on the left. For each patient's row, black boxes indicate regions where all genotyped SNPs show LOH in the tumor samples. Gray blocks indicate regions of uninformative SNPs in between observed regions of LOH. Unmarked areas of each sample indicate informative SNPs where no LOH was observed. The dotted lines highlight the region covered by *SOSTDC1*. We note that three samples (two Wilms and one RCC) show a large region of LOH that includes either the entire genotyped region (W-733 and W-8188) or a ~1 Mb region including *SOSTDC1 *(RCC-614). LOH does not appear to center around a particular gene. The genes within this region of interest code for the following proteins: transmembrane protein 195 (TMEM195); mesenchyme homeobox 2 (MEOX2); isoprenoid synthase domain containing (ISPD); sclerostin domain-containing protein (SOSTDC1); ankyrin repeat and MYND domain-containing protein 2 (ANKMY2); basic leucine zipper and W2 domain-containing protein 2 (BZW2); tetraspanin-13 (TSPAN13); anterior gradient protein 2 homolog precursor (AGR2); anterior gradient protein 3 homolog precursor (AGR3); aryl hydrocarbon receptor precursor (AHR); and sorting nexin-13 (SNX13).

To test whether the reduced SOSTDC1 expression could be attributed to genetic losses at 7p, we performed a SNP and sequencing analysis of *SOSTDC1 *in 25 pediatric and 36 adult renal cancers. In Wilms tumors, SNP genotyping over the 2.4 Mb region at 7p21.1 to 7p21.2 revealed LOH in three of the 25 tumors (Figure 2; patient numbers W-733, W-8188, and W-8194). These LOH-containing samples included a patient with hemihypertrophy being evaluated for Beckwidth-Wiedemann syndrome with a Stage II tumor that showed complete LOH at every informative SNP in the region (Patient W-733); a patient with a multifocal Wilms tumor also showing complete LOH at every informative SNP (W-8188); and a patient with anaplastic Wilms (W-8194), showing one instance of LOH at SNP rs6942413, near *MEOX2*.

Direct sequencing of the *SOSTDC1 *allele revealed one additional patient, W-8197, with one instance of LOH affecting the 3' untranslated region (UTR) in exon 5 of *SOSTDC1*; all other sequences in this patient showed no informative SNPs. Direct sequencing also confirmed that LOH directly affects *SOSTDC1 *in patients W-733 and W-8188, as every heterozygous SNP in the normal was lost in the tumor (Table 1). Patient W-8194 had no informative SNPs seen in the direct sequence of *SOSTDC1*, so it was not possible to ascertain whether this patient exhibited LOH at *SOSTDC1*. Sequence analysis revealed no mutations within known exons (3 and 5) or candidate exons (1, 2, and 4) of the remaining *SOSTDC1 *allele.

**Table 1:** Results of direct sequencing of SOSTDC1

Sample	Location	Informative SNPs without LOH	Normal	Tumor
RCC-129	End of Exon 1: rs35324397	Yes	A/G	G

RCC-614	Beginning of Exon 1: 16,536,670; 16,536,667 between rs10240242 and rs35324397	Yes	G/T, A/G	T, A

RCC-614	Beginning of Exon 1: 16,536,641 between rs10240242 and rs35324397	Yes	C/G	C

RCC-614	End of Exon 1: rs35324397	Yes	C/G	C

RCC-614	End of Exon 1: 5 bp downstream of rs35324397	Yes	A/G	G

RCC-635	Beginning of Exon 1: 16,536,641 between rs10240242 and rs35324397	Yes	C/G	C

RCC-737	Exon 5: 16,468,252 closest to rs6959246	Yes	G/T	T

W-733	Before Exon 1: rs7781903	No	C/T	C

W-733	Beginning of Exon 1: between rs10240242 and rs35324397	No	C/G	G

W-733	Beginning of Exon 2: rs7801569	No	C/T	C

W-8188	Beginning of Exon 2: rs7801569	No	C/T	C

W-8197	Exon 5: 16,468,252 closest to rs6959246	No	G/T	T

SNPs found in the direct sequences are summarized here. All other samples sequenced showed no LOH or other mutations. SNP location relative to sequenced exons and chromosome 7 base pair location is provided. The existence of heterozygous SNPs (informative, but with no LOH present) in the sample is shown via yes/no designation. RCC = adult renal carcinoma samples, W = pediatric Wilms tumors.

### Loss of heterozygosity at 7p21 in adult renal tumors

Three of the 36 adult patients samples analyzed showed LOH in the 2.4 Mb region of interest (Figure 2). Two of these patients had clear cell renal carcinoma (RCC-1 and RCC-614); while one had a less common oncocytoma (RCC-635). Patient RCC-614 showed LOH over much of the area, while RCC-1 and RCC-635 showed LOH at approximately 15-20% of informative SNPs. Direct sequencing of *SOSTDC1 *exons in adult tumors also showed LOH in patients RCC-614 and RCC-635 in several locations of exon 1 (Table 1). Additionally, patients RCC-129 and RCC-737 also showed LOH in one SNP each.

The adult tumors displaying LOH did so at some but not all loci, even within the *SOSTDC1 *gene itself. This is in contrast to what was observed within the Wilms tumors, where the samples with LOH displayed complete LOH at every heterozygous allele. Among all samples (adult and pediatric), LOH within *SOSTDC1 *was observed mostly in the putative exon 1, with no observed heterozygosity loss in the regions of the gene that are known to be transcribed. Whether adult or Wilms, for each SNP that showed LOH in more than one sample, the same allele was lost. For example, at the beginning of exon 1 (position 16,536,641) the G is absent from the C/G in RCC-614 and RCC-635 (Table 1).

### Impact of SOSTDC1 LOH on protein expression

We hypothesized that *SOSTDC1 *LOH might lead to decreased protein expression in the RCC and Wilms tumor samples. To address this possibility, the SOSTDC1 protein expression of tumor samples with and without LOH at *SOSTDC1 *was analyzed by immunohistochemistry. Antiserum from rabbits immunized with a peptide corresponding to the 18 C-terminal amino acids of the SOSTDC1 protein was used for this analysis. The antiserum has been used previously in an immunohistochemical application and additional characterization is included ([16]; see Additional file 4).

**Figure 3:**
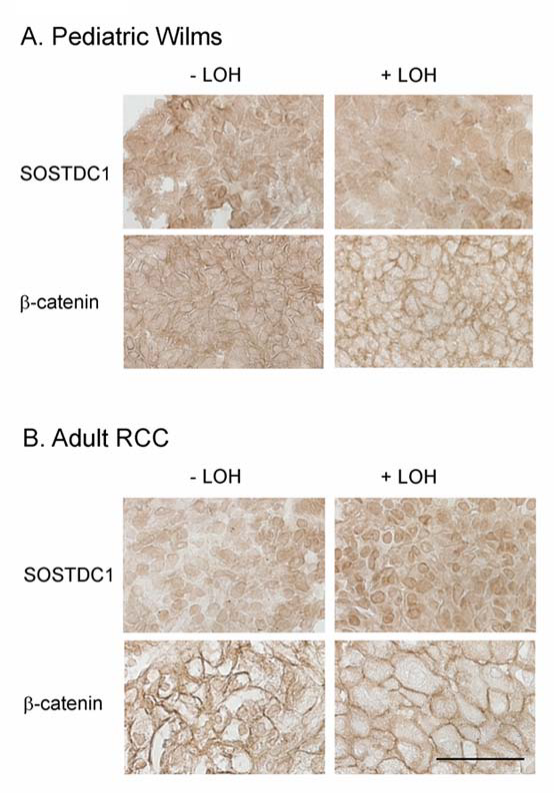
**Immunohistochemical analyses of SOSTDC1 and β-catenin protein levels and localization**. A) Pediatric Wilms tumor samples and B) adult renal cell carcinoma samples with and without *SOSTDC1 *LOH were stained with antibodies directed against SOSTDC1 and β-catenin. No consistent staining differences were observed between samples with LOH and those without. Representative images are shown. Scale bar = 50 μm.

When tumor samples were stained for SOSTDC1, the protein showed defined perinuclear and diffuse cytosolic localization in both adult and pediatric renal tumors. Representative images are shown in Figure 3. SOSTDC1 expression was not markedly reduced within tumor samples with *SOSTDC1 *LOH in either Wilms tumors or RCC [compare Wilms -LOH (W-8178) to Wilms +LOH (W-733) in Figure 3A and adult renal tumors -LOH (RCC-347) to +LOH (RCC-614) in Figure 3B]. Other samples with *SOSTDC1 *LOH similarly exhibited no observable variations in SOSTDC1 protein expression or localization. As the *SOSTDC1*-specific LOH in these samples was largely in the putative or regulatory exon 1 (Table 1), this observation is not necessarily unexpected.

### Effect of LOH at SOSTDC1 on Wnt signaling

Given that LOH at *SOSTDC1 *may lead to protein reductions that would be too subtle to be detected by immunohistochemistry and no obvious reductions in SOSTDC1 levels were observed in patient samples, we examined effects of LOH at *SOSTDC1 *on Wnt signaling. The likelihood that signaling might amplify the effects of SOSTDC1 variations increased the possibility for detection. We hypothesized that *SOSTDC1 *LOH would decrease the protein's abrogation of Wnt-induced signaling, resulting in increased β-catenin stability and/or nuclear localization.

To analyze the effect of *SOSTDC1 *LOH on cell signaling in pediatric Wilms tumors, patient samples with or without LOH were stained with a β-catenin-specific antibody. As shown in Figure 3A, the β-catenin localized largely to the cell periphery in the pediatric tumor samples. The LOH status of the samples did not correspond with obvious changes in β-catenin levels and localization [Figure 3A, compare -LOH (tumor W-8181) to the +LOH sample (W-733)].

Adult renal carcinoma samples with and without LOH at *SOSTDC1 *were also examined for changes in Wnt signaling via immunohistochemistry. As in the pediatric renal tumors, the β-catenin localized largely to the cell membrane. LOH-specific alterations in β-catenin were not evident in the adult renal cell tumors. [Figure 3B, compare the -LOH sample (RCC-377) to sample with *SOSTDC1 *LOH (RCC-1)]. Thus, in the patient samples we examined, *SOSTDC1 *LOH was not associated with consistent or strong changes in Wnt-induced signaling.

## Discussion

The frequency of deletions within the short arm of chromosome 7 in adult and pediatric renal tumors highlights the possibility that this region may contain genes that encode renal tumor suppressors. Evidence from Wilms tumors has narrowed the region of interest on chromosome 7 to a 2-Mb region within 7p21 that contains ten known genes, including *SOSTDC1 *[10]. Observations that SOSTDC1 is expressed in normal renal tissue and that its expression is decreased in renal cancer ([16]; Figure 1) coupled with this secreted protein's role in modulating the cancer-relevant BMP and Wnt signaling pathways, led us to hypothesize that LOH within the *SOSTDC1 *locus may contribute to renal tumor development.

We investigated the frequency of LOH within the *SOSTDC1 *gene in pediatric Wilms tumors and adult renal tumors. Overall, we observed LOH at the *SOSTDC1 *gene in 4/25 (16%) of Wilms tumor patients. This frequency is comparable to that of known Wilms tumor suppressors *WT1 *and *CTNNB1 *[30-32]. The rate of *SOSTDC1 *mutations observed in our studies was somewhat higher than that reported by Ohshima and coworkers (4/100;[10]). This disparity can potentially be attributed to sample size limitations and/or experimental variations. It should be borne in mind that current treatment of Wilms tumors sometimes involves pre-surgical chemotherapy or radiation. Differences in treatment status within the patient population may have effects on the resulting tissues used to obtain genomic DNA and thus the results of the LOH studies.

LOH in Wilms tumors appears to occur in large sections on the short arm of chromosome 7, as seen in patients W-733 and W-8188 (Figure 2). This is concordant with previous studies [4,10,33,34]. Notably, two patients (W-8194 and W-8197) showed examples of just one instance of LOH each. Due to distances between LOH markers for patient W-8194 (approximately 100 kb), and a lack of informative SNPs in *SOSTDC1*, it is unclear whether this region of LOH extends beyond the *SOSTDC1 *locus. Patient W-8197 showed one instance of LOH in the direct sequence. As no other informative SNPs were found within the direct sequence, this may represent either LOH affecting *SOSTDC1 *or a point mutation. It is noteworthy that tumor size, stage, histology, and treatment status varied among these patients.

We observed LOH affecting the *SOSTDC1 *locus at a frequency of 5/36 (14%) in adult RCC. In contrast to the observations within the Wilms tumors, the regions of LOH in adult RCC tumors were noncontiguous, as SNPs showing LOH were broken up by heterozygous alleles. Due to the high incidence of aneuploidy in these tumors, this phenomenon may be partially explained by chromosomal copy number variation. Indeed, multiple studies referenced in the Database of Genomic Variants show variations in copy number that affect parts of the 2 Mb region; including the area around *SOSTDC1 *[35,36].

We have previously reported downregulation of both the message (90% of patients) and protein encoded by *SOSTDC1 *in RCC-clear cell tumors [16]. To determine whether or not these observations could be attributed to LOH, we performed immunohistochemistry on the patient samples that had displayed LOH at *SOSTDC1*. We found that SOSTDC1 protein levels were comparable between samples that displayed LOH and those that did not (Figure 3), indicating that the instances of LOH observed in our patient samples were not associated with a detectable decrease in SOSTDC1 protein expression.

Considering previous observations that SOSTDC1 negatively regulates Wnt-induced signaling in renal cells, we also tested whether *SOSTDC1 *LOH corresponded to increased Wnt signaling in patient samples. To this end, immunohistochemical analyses were undertaken to compare SOSTDC1-relevant signaling between samples with and without LOH. This staining showed that LOH status did not consistently alter the levels or localization of β-catenin, a marker of Wnt pathway activation (Figure 3).

The observations that LOH at *SOSTDC1 *did not decrease SOSTDC1 protein expression or increase Wnt-induced signaling suggest that LOH may not be the key regulator of SOSTDC1 protein expression in pediatric and adult renal tumors. While LOH may play a role in the regulation of this locus in some patients, other mechanisms, including epigenetic regulation, must also be considered. For example, promoter methylation has been shown to have an important role in regulation of the *IGF2 *gene [37-39] and loci at 11p13 and 11p15 in Wilms tumors [16]. Improper splicing, a mechanism that contributes to dysregulation of the Wilms tumor suppressor gene *WT1*, might also contribute to the observed downregulation of SOSTDC1 in kidney cancer [37].

Although our limited sample size does not allow us to definitively refute the hypothesis that LOH is the primary regulator of SOSTDC1 in pediatric and adult renal tumors, we suggest that other modes of regulation must also be considered. Future experiments that investigate alternative regulatory mechanisms such as epigenetic silencing of *SOSTDC1 *may uncover more pertinent contributors to the reduced SOSTDC1 protein levels observed in renal cancer.

## Conclusions

This study investigates the role of *SOSTDC1*, a candidate renal tumor suppressor gene, in adult and pediatric renal tumors. We observed within an analysis of the Oncomine database that SOSTDC1 is expressed in normal renal tissue and that its expression is decreased in adult and pediatric renal cancer. When adult renal cell carcinoma samples were investigated for LOH within *SOSTDC1*, we found that LOH was present in five of 36 adult renal carcinoma samples and four of 25 Wilms tumors. This led us to investigate the possibility that *SOSTDC1 *LOH correlates with reduced protein levels or altered signaling. Our analyses did not reveal any consistent correlations between *SOSTDC1 *LOH and either SOSTDC1 protein levels or signaling. These findings point to the possibility that SOSTDC1 downregulation within adult and pediatric renal tumors may be attributable to a mechanism other than LOH, such as epigenetic silencing.

## List of abbreviations

The following abbreviations were used: AGR2: anterior gradient protein 2 homolog precursor; AHR: aryl hydrocarbon receptor precursor; ANKMY2: ankyrin repeat and MYND domain-containing protein 2; ATCC: American Type Culture Collection; BMP: bone morphogenetic protein; bp: base pairs; BZW2: basic leucine zipper and W2 domain-containing protein 2; CEU: Utah residents with Northern and Western European Ancestry; CHTN: Cooperative Human Tissue Network; DAB: 3,3'-diaminobenzidine; EDTA: ethylenediaminetetraacetic acid; GAPDH: glyceraldehyde 3-phosphate dehydrogenase; GST: glutathione S-transferase; IDT: Integrated DNA Technologies; ISPD: isoprenoid synthase domain containing; kb: kilobase pairs; LOH: loss of heterozygosity; Mb: megabase pairs; MEOX2: mesenchyme homeobox 2; RCC: renal cell carcinoma; SDS: sodium dodecyl sulfate; SNP: single nucleotide polymorphism; SNX-13: sorting nexin-13; SOSTDC1: sclerostin domain containing 1; TMEM195: transmembrane protein 195; TSPAN13: tetraspanin-13; UCSC: University of California, Santa Cruz; UEP: unextended primer; W: Wilms' tumor; Wnt: Wingless-Int; YRI: Samples from Yoruba descent Ibadan, Nigeria.

## Competing interests

The authors declare that they have no competing interests.

## Authors' contributions

KB performed the database interrogation and the *SOSTDC1 *LOH analysis and sequencing. KC carried out the sample staining and manuscript preparation. GH oversaw the *SOSTDC1 *LOH analysis and sequencing. AG assisted with the Wilms tumor tissue procurement. MW provided technical advice and interpretations for the immunohistochemistry results. JT aided in the *SOSTDC1 *LOH analysis and sequencing. FT assisted with the experimental design and interpretation. ST oversaw experiment planning, interpretation, and manuscript preparation. The final manuscript was read and approved by all authors.

## Supplementary Material

Additional file 1 **Map of the *SOSTDC1 *locus**. Arrows indicate the relative positions of designed primer pairs to potential regions of interest within the *SOSTDC1 *gene. The sizes of the known and putative exons are noted above the map; intron sizes are indicated below. The gene translation start codon is in exon 3 and the stop codon is in exon 5. All known coding sequences are contained within exons 3 and 5 (denoted by black boxes). Putative exons 1, 2, and 4 are highlighted by gray boxes. Data summarized from the Genome Browser hosted at UCSC. Click here for file

Additional file 2 **Primers for direct sequencing of *SOSTDC1***. Target exon, forward (F) and reverse (R) primer sequences, and amplicon sizes are shown. All primers designed to potential exons or regulatory regions of *SOSTDC1 *and were optimized for 60°C reaction temperatures. Click here for file

Additional file 3 **Primers for loss of heterozygosity analysis by single nucleotide polymorphism genotyping**. Sequences of the primers used for SNP LOH evaluation are shown. All primers designed for use on the Sequenom MassARRAY platform. The percentage of heterozygosity among informative SNPs within two populations from the International HapMap Project are listed. (CEU = Utah residents with Northern and Western European Ancestry; YRI = Samples from Yoruba descent Ibadan, Nigeria; UEP = unextended primer). Click here for file

Additional file 4 **Characterization of SOSTDC1-specific antiserum**. A) A renal cell carcinoma sample with LOH at the *SOSTDC1 *locus was treated with and without SOSTDC1 antiserum as an internal control to demonstrate effective SOSTDC1 detection. B) Increasing amounts of recombinant SOSTDC1 protein were gel-resolved and immunoblotted with SOSTDC1 antiserum. C) Proteins from the breast carcinoma cell line MDA-MB-231 and those from the breast epithelial cell line MCF10A were resolved and immunoblotted with SOSTDC1-specific antiserum in the presence or absence of competing peptide. The lack of banding in the presence of the immunizing peptide demonstrates antibody specificity. Glyceraldehyde 3-phosphate dehydrogenase (GAPDH) protein levels were used to verify loading. D) SOSTDC1 was purified from HEK-293 cells transiently transfected to express FLAG epitope-tagged SOSTDC1 protein. The coincident banding when membranes were probed with FLAG-specific antibody and SOSTDC1-directed antiserum verifies the specificity of the antiserum. Click here for file
