# Supplementary material for: Loss of heterozygosity and SOSTDC1 in adult and pediatric renal tumors
Source: J Exp Clin Cancer Res. 2010 Nov 16;29(1):147. doi: 10.1186/1756-9966-29-147 (PMC3002326; doi:10.1186/1756-9966-29-147)
Supplement: Additional file 2 — Primers for direct sequencing of SOSTDC1. Target exon, forward (F) and reverse (R) primer sequences, and amplicon sizes are shown. All primers designed to potential exons or regulatory regions of SOSTDC1 and were optimized for 60°C reaction temperatures. [file 1756-9966-29-147-S2.DOCX]

| **Primer**  **(Exon, #)** | **Sequence** | **Amplicon** |
| --- | --- | --- |
| 1-1 F | CCAGCCATTCTACCTCCAGG |  |
| 1-1 R | TGAAGTGTGTGCATTTTGTATTCA | 785 |
| 1-2 F | TGCATAGTGTTTGGGGTGG |  |
| 1-2 R | TGAAGTGTGTGCATTTTGTATTCA | 877 |
| 2-1 F | GGCAGTTCCCCTGCACAT |  |
| 2-1 R | GGCCTGAAGGGAGGTGAAG | 604 |
| 2-2 F | TGGTTTGACCAGTCCCCACT |  |
| 2-2 R | ATAGTTCTCCACACAATCTCCTCA | 656 |
| 3-1 F | TAGATTCAGGAAAGGAAATGGC |  |
| 3-1 R | ACTTACTGTTCCGATCCAGTCC | 572 |
| 3-2 F | TCCCACCCCTTCTCTGTGTT |  |
| 3-2 R | ATGGTCATTTTGCATGATTTTG | 680 |
| 3-3 F | ACACCTGAATGAACGCCAAACCTC |  |
| 3-3 R | TAGGGAAGAATGCCAACCTGCACA | 495 |
| 4 F | CTTACACAAATCTTTTGCCTCTCC |  |
| 4 R | ATCAGGAGTTTCACTTCATCTCTG | 520 |
| 5-1 F | CATGAAAGTGTCCCTATACTATCCA |  |
| 5-1 R | CTAACTCATGCTGTGCTTGCT | 597 |
| 5-2 F | GTACTGGAGCAGGAGGAGCT |  |
| 5-2 R | AGGAAGATCACTCATGGCTGC | 750 |
| 5-3 F | TCAGGACCTTCTTTGGGAATAG |  |
| 5-3 R | GGTCAAGACACCTTCTGATTGC | 810 |
| 5-4 F | CGCTTGGAATGGAATGCC |  |
| 5-4 R | AATGAGCAGCAGACTTGGCA | 668 |
| 5-5 F | CCTGCCAGTGCTCCCTAACT |  |
| 5-5 R | CATTCCAAGCGAGGGTCAG | 902 |
