# Supplementary material for: Loss of heterozygosity and SOSTDC1 in adult and pediatric renal tumors
Source: J Exp Clin Cancer Res. 2010 Nov 16;29(1):147. doi: 10.1186/1756-9966-29-147 (PMC3002326; doi:10.1186/1756-9966-29-147)
Supplement: Additional file 3 — Primers for loss of heterozygosity analysis by single nucleotide polymorphism genotyping. Sequences of the primers used for SNP LOH evaluation are shown. All primers designed for use on the Sequenom MassARRAY platform. The percentage of heterozygosity among informative SNPs within two populations from the International HapMap Project are listed. (CEU = Utah residents with Northern and Western European Ancestry; YRI = Samples from Yoruba descent Ibadan, Nigeria; UEP = unextended primer). [file 1756-9966-29-147-S3.DOCX]

| **Marker** | **% Informative** | | **Sequence** |
| --- | --- | --- | --- |
|  | **CEU** | **YRI** |  |
| rs4598143 | 53.4 | 64.5 | 1-ACGTTGGATGCAGTGTTTTTCTTTACAGAG  2- ACGTTGGATGGAGAACTCAATTTGCTTCCTC  UEP- TTGCTTCCTCTTGTTTATCCTTC |
| rs6944475 | 55 | 69.5 | 1- ACGTTGGATGGAGACTATATTTTCTAGCCCC  2- ACGTTGGATGGTTATTATCACTCACAGTGC  UEP- GGGAACAGTTCCGTAAACCTATCAAG |
| rs11982985 | 45.6 | 90.8 | 1- ACGTTGGATGGCATTACTTATCTGTAGTTTC  2- ACGTTGGATGACCAGAGCTGGCATTCTTTC  UEP- CTGTGTTTGCCCTCTCTTT |
| rs7783003 | 47.4 | 81.4 | 1- ACGTTGGATGGTCCGGATTCCATACTTTGC  2- ACGTTGGATGTCCCTGGTAACCTTACCTAC  UEP- GCACCTTACCTACAATTGTTTACATA |
| rs10950562 | 47.4 | 76.3 | 1- ACGTTGGATGATGGTACCAGCTCGTCTTTG  2- ACGTTGGATGCCTACCAACCAAAAAACGCC  UEP- AAACGCCTAGGATCAAAC |
| rs6946099 | 45.8 | 87.5 | 1- ACGTTGGATGGACAAACTCAGGTATTATG  2- ACGTTGGATGCGACCCCAATTACTTCCTAC  UEP- TGTCAGACTATATTTTCCTCTTCA |
| rs6942413 | 48.3 | 27.6 | 1- ACGTTGGATGCTCCCGAGAAAACTTGTTGG  2- ACGTTGGATGCCTCCCTCCCTCAATTTATG  UEP- CCCCCCTCAATTTATGAATTACAGAA |
| rs37410 | 50.8 | 17.5 | 1- ACGTTGGATGGAGTTCATAGTGAATGATTCC  2- ACGTTGGATGCCCCAAACCCAATAAACCAC  UEP- AACCACTAATCTGCTTTCAGTC |
| rs38176 | 53.3 | 95.8 | 1- ACGTTGGATGCTCAACGTTAAGCATGGTTC  2- ACGTTGGATGGAGCTCTGCAGAATGGAAAC  UEP- GAAACCCACTTCAAGTTGT |
| rs13231687 | 47.5 | 60 | 1- ACGTTGGATGAGTTGAACTGTAGCATCCGC  2- ACGTTGGATGCTGCGAATTCACAGCAGTAG  UEP- CAGCAGTAGGTTGGGAT |
| rs9648211 | 55 | 88.3 | 1- ACGTTGGATGTGTTGCCCTATCTCATAAGC  2- ACGTTGGATGCCTTTGCTAACCTTACGGAC  UEP- GGACCATTTCCCAATACTG |
| rs10277151 | 50 | 75.4 | 1- ACGTTGGATGCATTGCAGAATGTTCTCTCC  2- ACGTTGGATGCCATAATAAAGCTGGAAGAG  UEP- TTGGCTGGAAGAGTATTGACTCAG |
| rs7777150 | 53.4 | 70.8 | 1- ACGTTGGATGATAAAAGGGTGGTACAAAGG  2-ACGTTGGATGTCTTTACATCCCAGAGTAGG  UEP- TCCCAGAGTAGGAATCACCTAT |
| rs6963052 | 54.6 | 76.8 | 1- ACGTTGGATGCATGTCTAAACCACTGGAAA  2- ACGTTGGATGTAGCAGGTACACTTGCTCAC  UEP- CTGCTCACTGATATCCCTCTT |
| rs12699778 | 47.5 | 96.7 | 1- ACGTTGGATGGAAGAAAGGAATGGAAGCAG  2- ACGTTGGATGCTTCCTGGATCTTTGGTTGG  UEP- TTGGTTGGTTTCTAACATTGATT |
| rs2178598 | 46.6 | 47.1 | 1- ACGTTGGATGGGGTATCTCTGAGTTCTGTC  2- ACGTTGGATGGCCACAATAATCTCAACAGG  UEP- CTCAACAGGAAAGATTTCATATAA |
| rs2704674 | 52.5 | 46.7 | 1- ACGTTGGATGTGGAAGTTGTCCATGTGCTC  2- ACGTTGGATGTGGTAGACACAGAGATGCAC  UEP- CCAAGGAAGGGCTTGTTG |
| rs10270965 | 56.6 | 48 | 1- ACGTTGGATGCTATTTATAATGCAGAAACC  2- ACGTTGGATGTACAGGCATGAGCCATCGT  UEP- ATAGTTTTCCTGGTTTTTTTATATTG |
| rs6959566 | 45 | 23.3 | 1- ACGTTGGATGGGGACAGTGTAAAGCACAAT  2- ACGTTGGATGCTGGAGATAGTCTGACTAGC  UEP- TGTTTGACCTCAAGAAAAATT |
| rs1524362 | 54.4 | 55.9 | 1- ACGTTGGATGTCACTTTTGTCATGTCTTG  2- ACGTTGGATGTGTTAATAGGCCACATGACC  UEP- TCTGTCTGTAATTTTCCATGA |
| rs6968649 | 55 | 40 | 1- ACGTTGGATGGTATGGAGCAGAAGTAGAAG  2- ACGTTGGATGGCTTGCTAGGCTTTCCAAAC  UEP- CACCTGGCCTAGCCCTTTTGC |
| rs1524358 | 45.8 | 40.8 | 1- ACGTTGGATGTTCGTCTTCTCTTTCCCCTC  2- ACGTTGGATGGTTACTTAACCAAGGATTAGC  UEP- AACCAAGGATTAGCAGAAAAA |
| rs578621 | 49.2 | 62.5 | 1- ACGTTGGATGGGCGGAGTGATTCAAAATAG  2- ACGTTGGATGCAAAACAGTTTTGACAGGATG  UEP- CACAGTTTTGACAGGATGTTCTCAT |
| rs479202 | 53.9 | / | 1- ACGTTGGATGTGTCTTTGGCTTGGAAGTGG  2- ACGTTGGATGATGAATGTGAGAGTGGCAGG  UEP- CCAAATGCCTAGAGAGAA |
| rs818488 | 45.2 | 8.5 | 1- ACGTTGGATGTATGTCCCCTTGGGAATGTG  2- ACGTTGGATGCCTGGTGATTTGGATGCAAG  UEP- ATGCAAGCTTGGGCTTA |
| rs706059 | 46.6 | 5.3 | 1- ACGTTGGATGCCTGGCCTGTAGGCATTTTT  2- ACGTTGGATGGTACAGTGAAAAATACGAGAC  UEP- ACAAATACGAGACAAATGTGAGAGAC |
| rs12112188 | 47.3 | 86.7 | 1- ACGTTGGATGCCTTACACAGTAACAGGGAC  2- ACGTTGGATGAGCCCACCTGGGTAATAATC  UEP- GAGACCCTTAACCACAC |
| rs2389710 | 50.8 | / | 1- ACGTTGGATGGGCTGCAATAGATACACCAC  2- ACGTTGGATGGACATTTCCTCATTCCTCCC  UEP- CTCCTCATTCCTCCCATTATACT |
| rs860196 | 45.5 | 0 | 1- ACGTTGGATGGGCACTAATCCAGGAGTTTG  2- ACGTTGGATGTGCTATGACTTACTCTTTGG  UEP- TGACTTACTCTTTGGAACTAT |
| rs17137106 | 52.8 | 90 | 1- ACGTTGGATGGTTCCTATGTTATCTCTCTG  2- ACGTTGGATGTACCTATGTTTTTCTTCAC  UEP- GGTAGGTGGTACCAGGCTCA |
| rs7799920 | 50.8 | / | 1- ACGTTGGATGTTCCTGATGTTGGCAATTAG  2- ACGTTGGATGACGAAAAGCTGTTTCTTTG  UEP- GATGTTTCTTTGAATGATCAATAAGA |
| rs4719491 | 47.5 | 77.5 | 1- ACGTTGGATGCCTACATTCTTTTTACCCAG  2- ACGTTGGATGTTGGGATGGAGTGTTGAGTG  UEP- TGTCTGAAAGTTTTCTCTTTACTCTC |
| rs6957270 | 52.5 | 74.2 | 1- ACGTTGGATGAGACTTGGGAAAGCTAAGTG  2- ACGTTGGATGAGGAGAGGACTCTATGAGAC  UEP- CCAAACCTGAACTCTACCA |
| rs11971406 | 45.5 | 72.9 | 1- ACGTTGGATGCAGATCCTCAGATCATCTCC  2- ACGTTGGATGAAGGAGAAGCCAAGTAGAGG  UEP- AGGAACCATGGAGCCAAGT |
| rs12531256 | 52.5 | 70.3 | 1- ACGTTGGATGTTTGTGGGATTCAGCTGAAC  2- ACGTTGGATGGTTAGAAGTAAAACAGCGCC  UEP- CAGCGCCTGATTTGACC |
| rs687265 | 53.3 | 45.8 | 1- ACGTTGGATGCAACAAATCTAGGAGTTG  2- ACGTTGGATGCATCATTTCTAATTGTCTTA  UEP- TGATTAGTCTATTTAGTGGCCTAT |
| rs524715 | 45.7 | 70.3 | 1- ACGTTGGATGACAGTCCTTCTAACCTTCCC  2- ACGTTGGATGTAGGACTGGACCCTTAACTG  UEP- GTCAGGGTAGGCAAGAA |
| rs652187 | 55 | 20 | 1- ACGTTGGATGGGAGAACAAATAAACACTG  2- ACGTTGGATGTGGTCACTTCTCTCAATTGC  UEP- CTACTCATCATTACTGTTTTTAGT |
| rs9638747 | 48.3 | 59.1 | 1- ACGTTGGATGCTTAGGAATCCTCTTCTCGC  2- ACGTTGGATGACTTCCTACTTGAGAGACCC  UEP- TTGAGAGACCCCTAACATAG |
| rs6964052 | 55 | 84.2 | 1- ACGTTGGATGTTGACAAGCTAATCCATAG  2- ACGTTGGATGTATCTGTAGCCTTCTCTGGG  UEP- CCGAAATGTAGACTTCCTCTCAGTTAT |
| rs9785042 | 45.8 | 55.9 | 1- ACGTTGGATGGATTCTTTATCTGACATGCC  2- ACGTTGGATGGGAACAGAACAGAAAGCCCA  UEP- CAAGCCCAAAAATAAATCCACGCT |
| rs12699872 | 54.2 | 90 | 1- ACGTTGGATGCCCTCTCCCTCTCAAATATA  2- ACGTTGGATGGTGGATGGAATTATTCAGA  UEP- GATGGAATTATTCAGAAAGTTGAG |
| rs1562632 | 54.2 | 94.8 | 1- ACGTTGGATGTCTACTTGTGCCTAGATGCC  2- ACGTTGGATGACATCTTATTGCCAAGAGTG  UEP- GATTGCCAAGAGTGGCACCAGACC |
| rs7788582 | 54.3 | 45 | 1- ACGTTGGATGCAGAAGGGATTATCAAGGTG  2- ACGTTGGATGAAGACTTTAGTCCTTTCACC  UEP- AAATGACCACACTAGCCAAG |
| rs2192073 | 54.2 | 17.2 | 1- ACGTTGGATGTTGTCTTCTACAAGGCCTAT  2- ACGTTGGATGGGGAGGTTGAAGTGGATTAC  UEP- CCTACCCTGCTAGTGGGATTGTA |
| rs10235024 | 46.7 | 13.3 | 1- ACGTTGGATGTCTAAAAAGTAGCACTTTC  2- ACGTTGGATGGTTTTGGGAGGTTATTTGAG  UEP- TAAGGGAAGAATTAGCAATAGTTA |
| rs12672575 | 47.4 | 33.3 | 1- ACGTTGGATGTTGCTCACTTGCTTGCTCTC  2- ACGTTGGATGCCTCACTTTCTGGCCAAATG  UEP- GGCAACTTACAACATGG |
| rs10950672 | 45.8 | 61.7 | 1- ACGTTGGATGAGGAGATCGTCCTTGATAAC  2- ACGTTGGATGCTACTACCTCACAGAACAAG  UEP- GGTCTGGAGAAAGTTGGTAGCA |
| rs2723548 | 54.2 | 59.3 | 1- ACGTTGGATGCGTCATCCTCCATTACCTTG  2- ACGTTGGATGATGGGCGAGATGAGCAGAAA  UEP- GCGAGATGAGCAGAAAACAATA |
| rs2110013 | 48.3 | 17.9 | 1- ACGTTGGATGACAGCACTCTAGCCTGTGT  2- ACGTTGGATGGCTCTATTGTTTAGGATGAG  UEP- CTTCAGACAGGGTCTTG |
| rs1051603 | 45.8 | 1.7 | 1- ACGTTGGATGGACCAGATACAGATGAGAAG  2- ACGTTGGATGCTGCACTCTACAATTAACGTC  UEP- ATTAACGTCTCCAAAAGTTAATA |
