# Supplementary figures and images for: Loss of heterozygosity and SOSTDC1 in adult and pediatric renal tumors
Source: J Exp Clin Cancer Res. 2010 Nov 16;29(1):147. doi: 10.1186/1756-9966-29-147 (PMC3002326; doi:10.1186/1756-9966-29-147)

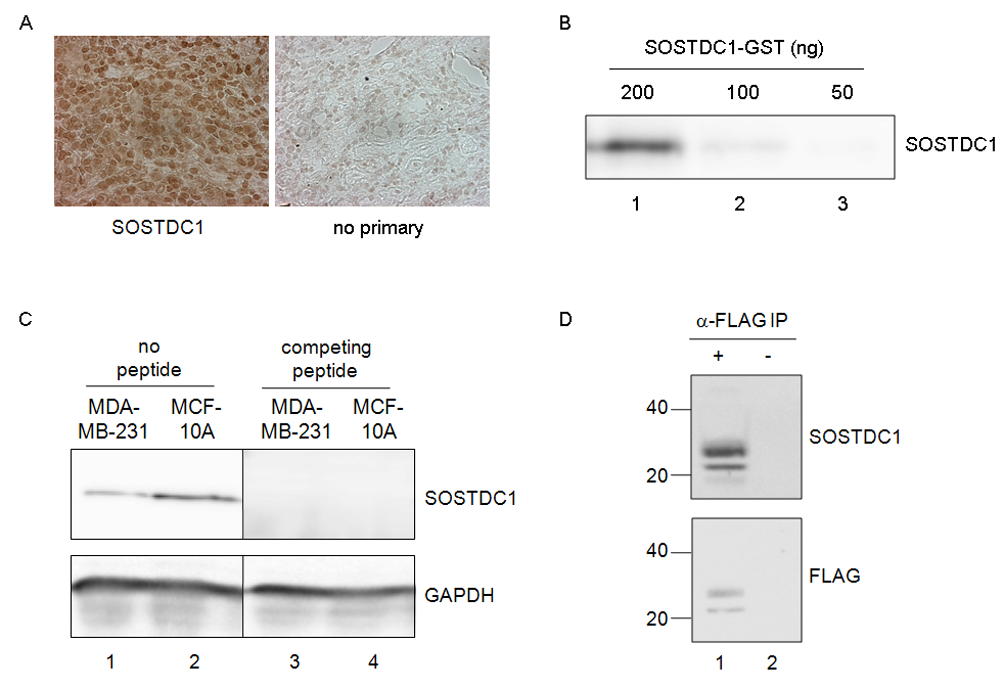

Supplement: Additional file 4 — Characterization of SOSTDC1-specific antiserum. A) A renal cell carcinoma sample with LOH at the SOSTDC1 locus was treated with and without SOSTDC1 antiserum as an internal control to demonstrate effective SOSTDC1 detection. B) Increasing amounts of recombinant SOSTDC1 protein were gel-resolved and immunoblotted with SOSTDC1 antiserum. C) Proteins from the breast carcinoma cell line MDA-MB-231 and those from the breast epithelial cell line MCF10A were resolved and immunoblotted with SOSTDC1-specific antiserum in the presence or absence of competing peptide. The lack of banding in the presence of the immunizing peptide demonstrates antibody specificity. Glyceraldehyde 3-phosphate dehydrogenase (GAPDH) protein levels were used to verify loading. D) SOSTDC1 was purified from HEK-293 cells transiently transfected to express FLAG epitope-tagged SOSTDC1 protein. The coincident banding when membranes were probed with FLAG-specific antibody and SOSTDC1-directed antiserum verifies the specificity of the antiserum. [file 1756-9966-29-147-S4.TIFF]
